# No symphony without bassoon and piccolo: changes in synaptic active zone proteins in Huntington’s disease

**DOI:** 10.1186/s40478-020-00949-y

**Published:** 2020-06-03

**Authors:** Ting-Ting Huang, Ruben Smith, Karl Bacos, Dong-Yan Song, Richard M. Faull, Henry J. Waldvogel, Jia-Yi Li

**Affiliations:** 1 Institute of Neuroscience, College of Life and Health Sciences, Northeastern University, 110819 Shenyang, P. R. China; 2 Department of Neurology, Skåne University Hospital, Lund University, Lund, Sweden; 3 Epigenetics and Diabetes Unit, Department of Clinical Sciences, Lund University Diabetes Centre, CRC 91:12, Jan Waldenströms gata 35, 21428 Malmö, Sweden; 4 Centre for Brain Research and Department of Anatomy and Medical Imaging, Faculty of Medical and Health Sciences, The University of Auckland, Auckland, 92019 New Zealand; 5 Department of Experimental Medical Science, Neuronal Plasticity and Repair Unit, Wallenberg Neuroscience Center, BMC A10, 22184 Lund, Sweden; 6 Institute of Health Sciences, China Medical University, 110112 Shenyang, P. R. China

**Keywords:** Huntington’s disease, Active zone proteins, Synaptic dysfunction, Protein aggregation

## Abstract

Prominent features of HD neuropathology are the intranuclear and cytoplasmic inclusions of huntingtin and striatal and cortical neuronal cell death. Recently, synaptic defects have been reported on HD-related studies, including impairment of neurotransmitter release and alterations of synaptic components. However, the definite characteristics of synapse dysfunction and the underlying mechanisms remain largely unknown. We studied the gene expression levels and patterns of a number of proteins forming the cytoskeletal matrix of the presynaptic active zones in HD transgenic mice (R6/1), in hippocampal neuronal cultures overexpressing mutant huntingtin and in postmortem brain tissues of HD patients. To investigate the interactions between huntingtin and active proteins, we performed confocal microscopic imaging and immunoprecipitation in mouse and HEK 293 cell line models. The mRNA and protein levels of Bassoon were reduced in mouse and cell culture models of HD and in brain tissues of patients with HD. Moreover, a striking re-distribution of a complex of proteins including Bassoon, Piccolo and Munc 13–1 from the cytoplasm and synapses into intranuclear huntingtin aggregates with loss of active zone proteins and dendritic spines. This re-localization was age-dependent and coincided with the formation of huntingtin aggregates. Using co-immunoprecipitation, we demonstrated that huntingtin interacts with Bassoon, and that this interaction is likely mediated by a third linking protein. Three structural proteins involved in neurotransmitter release in the presynaptic active zones of neurons are altered in expression and that the proteins are redistributed from their normal functional site into mutant huntingtin aggregates.

## Introduction

Huntington’s disease (HD) is a fatal autosomal dominant neurodegenerative disorder, caused by a CAG-triplet repeat expansion in the gene *IT15* encoding the protein huntingtin [9]. The main symptoms and signs of HD are cognitive deterioration and personality changes combined with involuntary choreatic movements. The mechanisms by which the mutated protein causes disease remain largely unknown. Cell death is the most pronounced in the striatum and in the cerebral cortex [49], but neurodegeneration has also been reported in the hypothalamus [18, 19, 32]. Another feature of HD is the formation of intranuclear and cytoplasmic inclusions largely composed of mutant huntingtin [4, 6]. Normal huntingtin is proposed to play a role in intracellular transport [27], gene transcription [36, 42], neurotransmitter synthesis, release and vesicle endocytosis [23, 39].

The R6 transgenic mice were generated by Mangiarini and collaborators [26]. The R6/1 mice show the first overt motor symptoms at an age of 16 weeks and decline in motor and cognitive function until the end stage at 40 weeks of age [10]. Neuronal cell death is not widespread, although some cells are lost in the hypothalamus [32]. We have previously shown that the SNARE proteins and Rabphilin 3A, both of which are involved in exocytosis and transmitter release, are affected in the R6/1 mouse model and in brain tissue from HD patients [39, 40]. Moreover, synaptic alterations, especially diminished exocytosis and compromised transmitter release, were also observed in other HD models [3, 12, 13, 33, 35, 48]. This led us to propose a synaptic defect being a possible culprit in HD pathogenesis [23, 39, 45]. Moreover, we have also demonstrated progressive impairment of dendritic spine dynamic, particularly affecting spine stability and survival in the somatosensory cortex of the R6/2 mouse strain [28, 29]. In *Huntingtin* CAG knock-in mice, reduced synapse density, accompanied with decreased striatal medium spiny neurons and striatal volume, implies dysfunction of synaptic plasticity [16]. Similarly, in ex vivo preparations of the skeletal muscle of R6/2 mice, although no clear denervation was exhibited, a reduction in the size of synaptic vesicle pool was observed in R6/2 mice compared to the WT mice. Possibly this reduction was caused by impairment of vesicle mobilization in neuromuscular junctions [15].

In order to elucidate if there was further synaptic dysfunction in HD model mice and patients, we now focused on a set of proteins that form the cytoskeletal matrix of the active zone (CAZ; for reviews see [8, 24, 34, 41, 53]). The CAZ defines the areas of the presynaptic compartments, particularly in the domains, where regulated neurotransmitter release occurs. At present, five proteins with an almost exclusive distribution to the presynaptic CAZ have been described. Bassoon and Piccolo, two large structural proteins (430 and 520 kDa, respectively), are believed to act as anchoring proteins [53]; RIM1, a Rab3a interacting protein, makes a link between the CAZ and the synaptic vesicles. RIM1 binds both ERC/CAST and Munc 13–1 [31]. Munc 13–1 interacts with the other CAZ proteins and syntaxin 1a tightly regulating transmitter release. ERC/CAST is a protein interacting with RIM1, Bassoon and Piccolo [24, 30, 47, 53]. Bassoon was first described as a CAG-containing protein, highly expressed in the cerebral cortex and the hippocampus and localized to the presynaptic active zone [44]. It is considered essential for normal retinal ribbon synapse formation [5, 43]. Bassoon knockout animals show a normal morphology of the hippocampal synapses, but an increased number of inactive synapses. A high proportion of these animals develop epileptic seizures and die within 6 months [1]. Bassoon and Piccolo are also present in the Golgi apparatus, where they are packaged and transported together to the presynaptic sites [7]. A close association of the CAZ proteins has been further corroborated by co-immunoprecipitation experiments [31].

In the present study, we have investigated the CAZ and demonstrated that the levels of the majority of CAZ and CAZ-associated proteins are not altered in the R6/1 animal model of HD. However, the levels of Bassoon mRNA and protein were decreased in a cell model, the R6/1 mouse model and in postmortem brain tissues from HD patients. With increasing age, the proteins appeared to be recruited into the mutant huntingtin aggregates in R6/1 mice and in brains of HD patients. We showed by immunoprecipitation that this is due to an interaction between huntingtin and bassoon. The data suggest that reduction of functional active zone proteins, accounted for by down-regulated gene transcription, reduced protein synthesis, and active sequestration of these proteins into mutant huntingtin aggregates, may negatively impact normal synaptic function.

## Materials and methods

### Human tissues

We obtained human brain samples from the New Zealand Neurological Foundation of New Zealand Human Brain Bank, Auckland, New Zealand. Ethical approval was granted from the University of Auckland Human Participants Ethics Committee in Auckland, New Zealand and at the Regional Ethical Review Board, Lund, Sweden (2017/12). Brains from control patients were macroscopically and microscopically confirmed not to suffer from brain disease. (See Table 1 for further details on sex, age, grade and post-mortem interval and the HD cases were graded by an independent neuropathologist using classic Vonsattel grading criteria.)

**Table 1:** Samples used for immunohistochemistry

*Case*	*Gender/Age*	*CAG-number*	*Vonsattel* *Grade*	*Post-mortem interval (h)*	*Region*
H109	M/81	15/18	N/A	7	BA9
H114	M/42	N/A	N/A	14	SFG
H152L	M/79	17/28	N/A	18	SFG
H164	M/73	17/23	N/A	13	SFG
HC92	M/72	17/41	1	5	SFG
HC82	M/74	15/42	2	16	SFG
HC96	F/39	22/53	3	20	SFG
HC102	M/64	17/42	3	10	SFG
HC104	M/40	18/51	3	15	SFG
HC77	F/53	16/55	4	9	SFG
HC87	M/45	21/50	4	18	SFG
HC109	F/59	23/47	4	7	SFG

*BA9* Brodmann area 9; *SFG* superior frontal gyrus; *N/A* not available

### Animals

The transgenic R6/1 mice in this study were purchased from Jackson laboratories (Jackson Laboratories, Bar Harbor, ME, USA) and bred with CBAxC57BL/6 wildtype (WT) females. A polymerase chain reaction was performed to genotype the mice [26]. The mice were housed under a 12-h light–dark cycle with food and water ad libitum. All the work involving animals were approved by the Ethical Committees for the use of laboratory animals at Lund University, Sweden and at Northeastern University, China.

### Western blotting

We dissected cortical samples from 7, 16 and 40-week-old mice. The tissues were homogenized on ice in buffer (2 mM EDTA, 4 mM HEPES, Protease inhibitor cocktail (Sigma, Stockholm, Sweden), pH 7.4) with 12 strokes, using a Dounce homogenizer. The homogenate was spun at 1000 x g for 10 min and the supernatant analyzed. After determining the protein concentration with a BioRad kit (BioRad, Hercules, CA), 1% SDS (final concentration) was added. Samples were diluted to 1 mg/ml in 3x Laemmli buffer containing β-mercaptoethanol (3% final concentration), after which they were heated to 95 °C for 5 min, and then kept at − 20 °C. 20 μg proteins were separated on 5% or 4–15% gradient SDS-PAGE gels, followed by immunoblotting onto PVDF membranes (GE Healthcare, Uppsala, Sweden). Due to the high MW of Bassoon a low methanol (5%), SDS-containing (0.01%) blotting buffer was used (pH 8.3). The membranes were incubated with the primary antibodies in 0.05% Tween-20 in PBS. Primary antibodies used were: Bassoon (Nordic BioSite SAP7F407, mouse, 1:1000); ERC, RIM1, Mint1 and CASK were all kind gifts from T. Südhof, (Stanford University, USA); Munc 13–1 (Synaptic Systems #126102, 1:1000); GAPDH (Chemicon mAB374, mouse, 1:10000). After rinsing, secondary antibodies (goat anti-mouse or donkey anti-rabbit IgG) conjugated with HRP (GE healthcare) were used; the signals were detected using ECL plus (GE Healthcare). ECL exposed films were scanned and the intensities of the bands were examined using ImageJ.

### Reverse transcription-PCR

Brains from 40w old R6/1 and WT animals were dissected and were quickly frozen in dry ice. Brains were kept at − 80 °C until used. The brains were semi-thawed and sectioned into ~ 2 mm sections on ice. The cortex was quickly dissected out and homogenized in 500 μl Diethyl pyrocarbonate (DEPC)-PBS: nucleic acid purification lysis solution (1:1) (Applied Biosystems, Foster City, USA). RNA was purified from the homogenate using a HT6100 RNA preparation station (Applied Biosystems). RNA (0.3 μg) was reverse transcribed with the Advantage™ RT-for-PCR Kit (BD Biosciences, Palo Alto, USA), using random hexamer primers, according to the manufacturer’s instructions. Synthesized cDNA corresponding to 75 ng was mixed with DEPC-H_2_0 and 2x TaqMan® Universal PCR Master Mix (Applied Biosystems). Samples were run on an ABI PRISM® 7900 HT Sequence Detection System (Applied Biosystems). The assays Mm00464452_m1 (mouse Bassoon) and 4,342,379-18S (18S RNA) were used. *n* = 9 per genotype.

### Immunohistochemistry

Animals were anesthetized and perfused transcardially with 4% paraformaldehyde (PFA). The brains were dissected and post-fixed in PFA for 24 h, before rinsing in 0.2 M phosphate buffer with 20% sucrose. The brains were sectioned into 30 μm sections in a cryostat. Primary antibodies used: Rabbit anti-Bassoon (Synaptic Systems #141002, 1:1000), mouse anti-huntingtin (Chemicon, clone mEM48, 1:500), rabbit anti-Piccolo (Synaptic Systems #142002, 1:1000). In brief, after blocking with normal sera from the species in which the secondary antibodies were produced, the sections were incubated with the primary antibodies overnight at room temperature. Followed by rinses and 2 h at room-temperature in Cy3−/Texas red-, Cy2−/FITC- and or Cy5-conjugated secondary antibodies (Jackson lab). In control sections (omitting the primary antibodies), specific immuno-fluorescence was never observed. The sections were viewed in a confocal laser-scanning microscope (Leica Microsystem, Stockholm, Sweden).

Brain blocks from the superior frontal gyrus of human postmortem tissues were serially sectioned in a cryostat (40 μm in thickness). For diaminobenzidine (DAB; Vector Lab. Inc., Burlingame, CA)-staining free floating sections were quenched in 3% H_2_O_2_ and 10% methanol for 15 min before blocking (5% normal horse or goat serum, 0.3% Triton-X-100 in 0.1 M PBS, pH 7.4) for 1 h. Primary antibody incubations (in 2% serum, 0.3% Triton-X-100) were carried out over night at room temperature. Primary antibodies used were rabbit Bassoon and mouse EM48 (see details above). After rinsing the sections were incubated in secondary antibody (Biotinylated Horse-anti-Mouse or Goat-anti-rabbit, 1:200, Jackson Lab, West Grove, PA), ABC-solution (Vector Lab) and finally DAB for 40 s. Positive signal was assessed from pictures obtained using a ScanScope CS (Aperio, Vista, CA).

For determining general staining intensity in human sections, the slides were scanned using a DuoScan f40 scanner (Agfa, Mortsel, Belgium) and the staining intensity analyzed in the cortical grey matter using the ImageJ software.

For double labeling with fluorescent secondary antibodies rabbit Bassoon, rabbit Piccolo, rabbit Munc 13–1 (Synaptic Systems, #126102; 1:1000) or rabbit SNAP-25 (Synaptic Systems, #111002; 1:1000) were combined with mouse EM48. Cy-2 or Cy-3 labeled secondary antibodies (Jackson Lab, West Grove, PA) were used. To block autofluorescence the sections were incubated in 5 mM CuSO_4_ in 50 mM ammonium acetate (pH 5.0) for 90 min prior to mounting.

### Immunoprecipitation

Mouse telencephalic samples of 40-week-old R6/1 and WT mice were dissected, and homogenized on ice in homogenizing buffer as described previously. Each homogenate was centrifuged at 1000 x g for 10 min; supernatant (S0) was collected. New homogenizing buffer was added to the pellet and the centrifugation was repeated. The supernatants were pooled and centrifuged once again at 1000 g for 10 min, whereupon a cleaned supernatant (S1) was collected. Protein concentrations of the samples were determined using the Pierce BCA protein assay reagent kit (Pierce, Rockford, IL, USA), and samples were diluted to 1 μg/μl in immunoprecipitation buffer containing 1 mM EDTA, 1% Nonidet P40, 1% protease inhibitor cocktail (Sigma P 8340) in PBS pH 7,4. 500 μl of each sample was pre-cleared using 50 μl of washed protein-G agarose (Upstate, Temecula, CA) for 30 min and incubated in 3 μg of mouse monoclonal anti-huntingtin antibody (MAB5492, Chemicon) at 4 °C overnight. The antibody-protein complex was captured by adding 20 μl of washed protein-G agarose and incubating for 2 h at 4 °C. A flow-through sample was collected after the immunoprecipitation, and the bead-antibody-protein complex was washed three times in immunoprecipitation buffer. The proteins were eluted and denatured by addition of 3X Laemmli buffer with 10% β-mercaptoethanol, and heating to 95 °C for 10 min. The samples were analyzed by SDS-PAGE/western blotting as described above.

HEK 293 cells immunoprecipitations were carried out as previously described [31]. Cells were cultured in DMEM containing 1% L-glutamine, Penicillin/Streptomycin and 10% fetal bovine serum at 37 °C in 5% CO_2_. 2 × 10^6^ cells were seeded onto Poly-L-Lysine-coated P100 dishes and let to attach overnight. The cells were double-transfected with Bassoon-GFP and HA-tagged-17Q- or 69Q-huntingtin using Lipofectamine 2000 (Invitrogen). Twenty-four-hour post transfection the cells were rinsed once in PBS before being scraped off in 1 ml of Triton-X-lysis buffer (20 mM Tris-HCl, pH 7.5, 100 mM NaCl, 0.5 mM EDTA, 1 mM DTT, 1% [wt/vol] Triton X-100, 1x Protease inhibitor cocktail (Sigma, P-8340)). Immunoprecipitations were carried out on the lysed cells using 1.5 μg of either GFP (Clontech 8362–1, Palo Alto, USA) or HA (ab 9110, Abcam, Cambridge, UK) as described above.

### Neuronal cultures and immunocytochemistry

We bred C57BL/6 males and females to generate pups. Primary neuronal cultures were prepared from the hippocampus of P0 (born within 24 h) pups as described previously [28]. At 7 days in vitro (DIV), hippocampal neurons were transfected with plasmids containing exon1 of huntingtin with either 17 (short-Q huntingtin) or 69 (long-Q huntingtin) glutamine residues under the control of a cytomegalovirus (CMV) promoter, with co-transfection with a plasmid expressing actin-GFP to label the postsynaptic compartments. At 11–12 DIV, neuronal cultures were fixed with 4% PFA for 15 min at 37 °C, permeabilized with 0.1% Triton X-100 for 3 min, and rinsed 3 times with PBS, blocked with 10% normal goat serum in PBS for 1 h, incubated with primary antibodies (Rabbit anti-Bassoon (Synaptic Systems, #141002, 1:500), mouse anti-huntingtin (Chemicon, clone mEM48, 1:500), rabbit anti-Piccolo (Synaptic Systems, #142002, 1:500), rabbit anti-Munc 13–1 (Synaptic Systems, #126102; 1:500)) at 4 °C overnight. After washing with PBS 3 times, the coverslips were incubated with goat anti-rabbit/mouse Alexa Flour®594 secondary antibody, 1:200 (Abcam). The coverslips were washed with PBS 3 times and then mounted with anti-fading mounting medium. Fluorescent images were acquired with a confocal laser scanning system (Leica confocal microscope) at 63x objective and 1x digital zoom.

For the cell culture experiments each protein analysis, *N* = 56–70 random neuronal segments (at high magnification), which derived from 19 to 23 neurons obtained from 3 technical replicates for each genotype (6–8 neurons each genotype). All types of spines, regardless of their shapes (mushroom or stubby spines) were included in the analyses.

### Statistics

To compare the protein level in R6/1 mice and WT mice, we used unpaired *t* test. To compare the protein amounts in R6/1 mice at different ages, we used a non-parametric Kruskal Wallis test, followed by a Mann-Whitney U test post hoc. We assessed the mRNA levels using the non-parametric Wilcoxons Signed Rank test with transgenic and control animals paired within the different experiments. To correlate changes in Bassoon intensity in human tissue of different grades, we used the non-parametric Jonckheere-Terpstra Test. To investigate the expression of Bassoon, Piccolo and Munc13–1 protein in the hippocampal neurons, used one-way ANOVA test, followed by a Tukey’s multiple comparisons post-tests. We considered statistical significance to be reached when *p* < 0.05.

## Results

### Expression of CAZ and CAZ associated proteins in the R6/1 mouse model

**Fig. 1:**
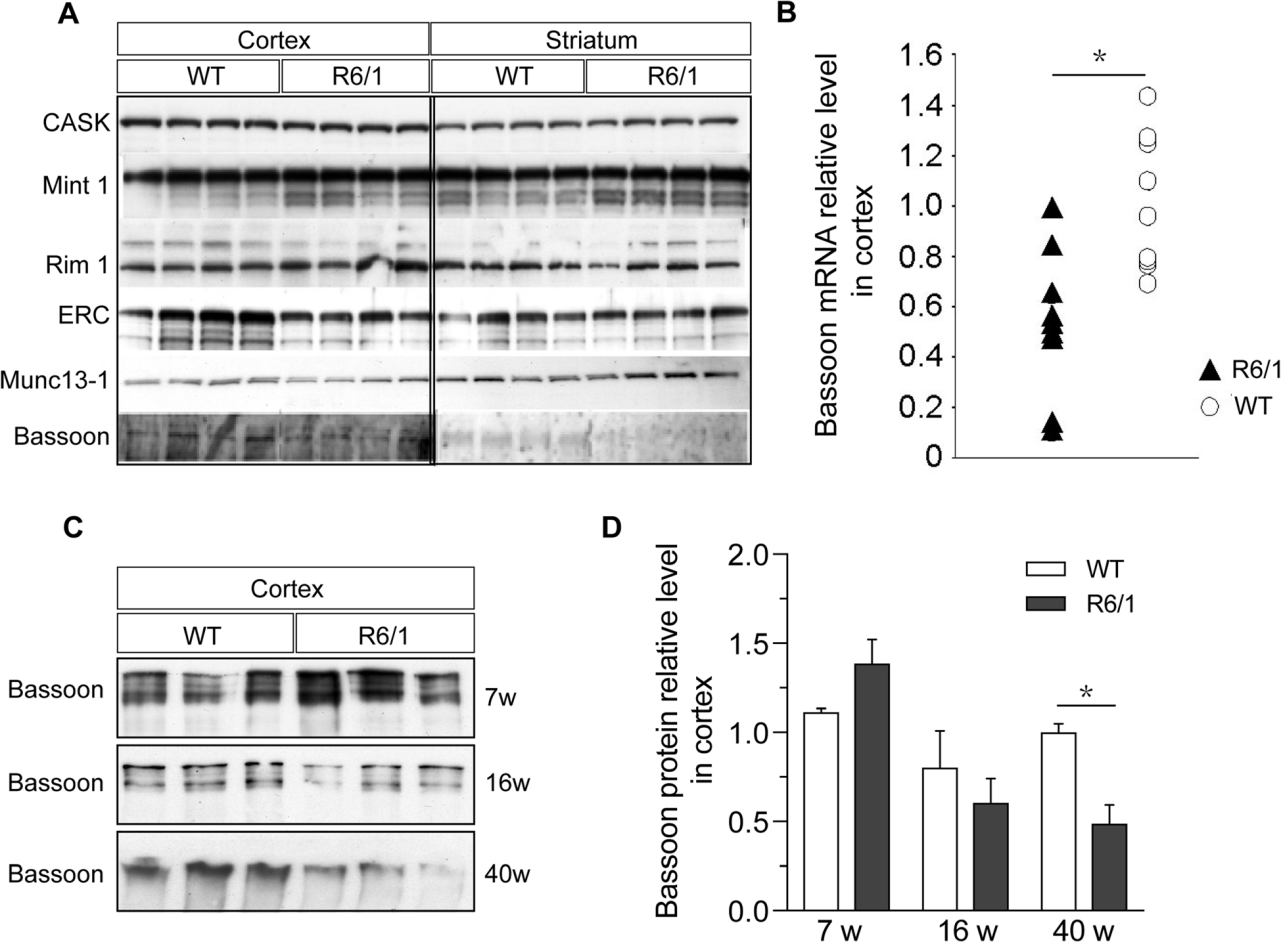
Protein levels and mRNA levels of active zone proteins and Bassoon. **a** Western blots showing the expression of CASK, Mint1, Rim1, ERC and Munc 13–1 Bassoon in the cortex and the striatum of 40w old R6/1 and WT mice. **b** Bassoon mRNA levels in 40w old R6/1 and WT mouse cortex. * *p* < 0.05, *n* = 9 / genotype. **c** Representative Western blot of Bassoon at 7, 16 and 40 weeks. **d** Bassoon protein levels in R6/1 and WT mice at 7, 16 and 40 weeks of age. *p < 0.05 *n* = 4 / genotype and age

We have previously studied the expression of a number of proteins involved in exocytosis in animal models of HD [39] and in samples from HD patients and control subjects [40]. A number of newly identified proteins forming the structural cytomatrix where exocytosis takes place were recently described [8, 24, 34, 41, 53]. To find out whether the proteins making up the cytomatrix of the active zones are affected by mutant huntingtin, we studied the expression levels of four of the active zone proteins (Bassoon, ERC, RIM1 and Munc 13–1) and two associated proteins (CASK and Mint 1; Fig. 1a) in the R6/1 mouse model at the late stage of HD (40 weeks old) and WT mice as control. At this age the R6/1 mice exhibit an overt phenotype, including motor and cognitive deficits with a substantial reduction of body weight (Mangiarini et al., 1996). The levels of the majority of active zone and active zone related proteins were not affected (no significant difference shown on quantification) in the brain of R6/1 mice (Fig. 1a, Supplement Fig. 1). However, we observed a progressive decrease in the levels of Bassoon and Munc13–1 in the R6/1 mice compared with WT ones (Unpaired *t* test, *p* = 0.02, *p* < 0.0001, *p* = 0.0001; Fig. 1a, Supplement Fig. 1E, F). Bassoon was reduced to 48.8 ± 10.5% in the cortex of R6/1 animals at 40 weeks of age (Mann-Whitney, *p* = 0.029, Fig. 1c, d). Similarly, we found that the mRNA levels of Bassoon in 40 weeks R6/1 cortex were decreased (median 52.4%, range 10.4–98.8%; *p* = 0.01, Wilcoxons signed rank test, Fig. 1b). Bassoon were both affected in the cortex and the striatum (Fig. 1a, Supplement Fig. 1F), suggesting these two regions might share similar pathology of Bassoon. Altogether, these results indicate the active zone proteins, i.e. Bassoon and Munc13–1, are down regulated in the cortex of HD mice, suggesting that the reductions are not mainly due to a general synaptic loss as other synaptic proteins are not reduced to a similar extent (Fig. 1a).

### Mutant huntingtin induces loss of active zone proteins and mature spines in primary neurons

**Fig. 2:**
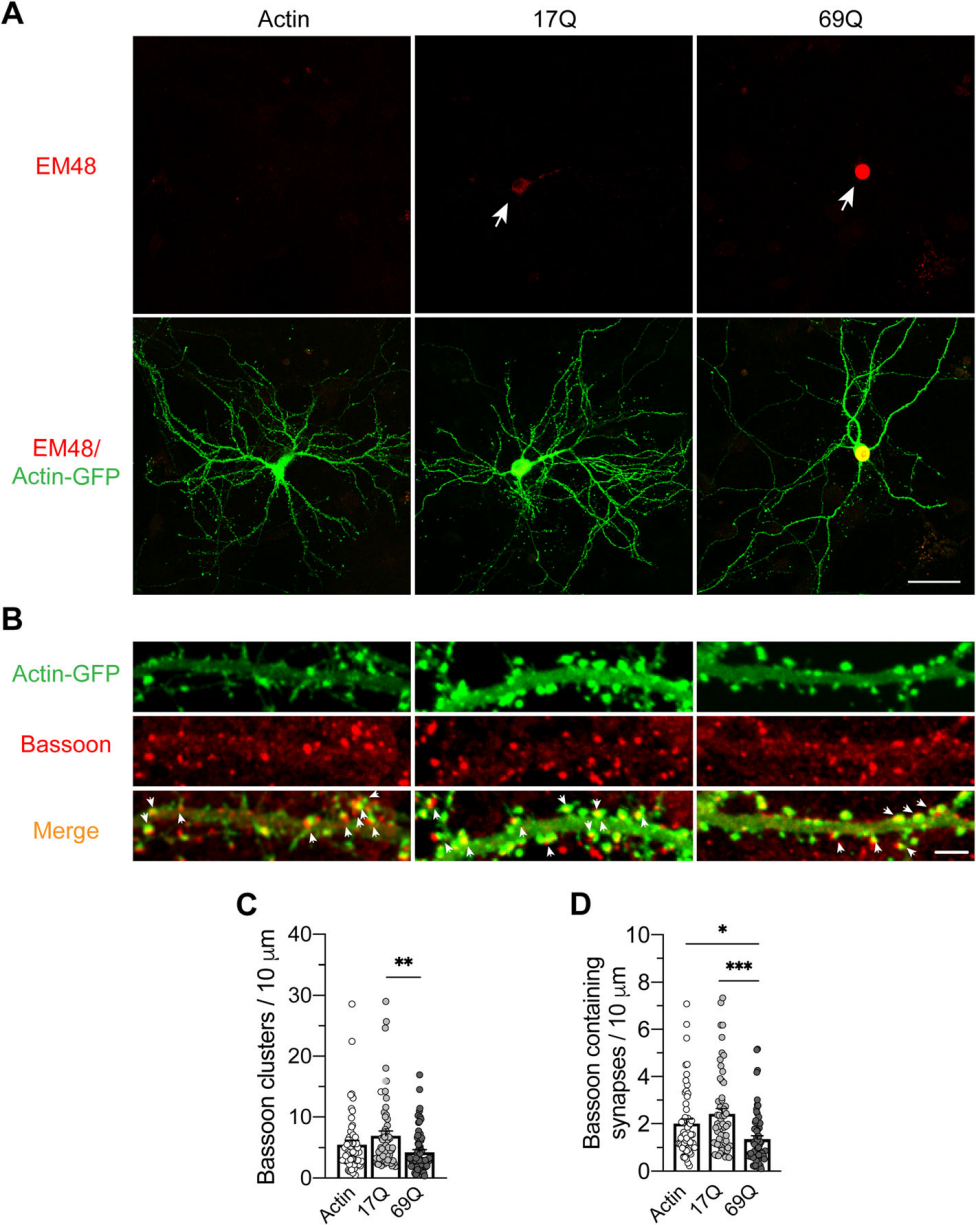
Primary neurons expressing mutant huntingtin protein (69Q) down-regulated the Bassoon protein expression. **a** Immunofluorescence of the huntingtin protein levels in GFP labeled neurons. Mutant huntingtin (69Q) expression induced the huntingtin aggregations (arrow) located in nucleus (scale bar = 75 μm). **b** Mutant huntingtin (69Q) caused the reductions of the linear densities of Bassoon clusters, leading to Bassoon containing synapse loss (arrowheads) in the dendrites (Scale bar = 5 μm). The quantifications of the linear densities of Bassoon (**c**), and Bassoon containing mature synapses (**d**). *p < 0.05, **p < 0.01, ***p < 0.001, *n* = 58–68 segments/group, *N* = 20–22 cells/group, triplicate individual experiments, one way ANOVA test, followed by a Tukey’s multiple comparisons post-test

To determine effects of mutant huntingtin on pre- and post-synaptic compartments, which were defined by immunopositive profiles stained with different active zone proteins and dendritic spines, respectively, we transfected primary neurons from WT mice with either short-Q huntingtin (huntingtin exon 1 with 17 glutamine residues; 17Q) or with long-Q huntingtin (huntingtin exon 1 with 69 glutamine residues; 69Q). Neurons were co-transfected with actin-GFP to mark the dendrites spine. We identified huntingtin aggregates using the EM48 antibody, specifically raised against aggregated huntingtin. The 17Q was diffusely present in the cytoplasm of the cell whereas the 69Q was only present in one large seemingly intranuclear aggregate, indicating the cell model was efficient to overexpress huntingtin and led to aggregation (Fig. 2a). We next immunostained actin-GFP-labeled neurons with antibodies against active zone proteins (Bassoon, Piccolo, or Munc13–1) to investigate the effect of mutant huntingtin on the expression level of active zone proteins. We observed significantly reduced levels of Bassoon and Munc13–1 proteins in neurons overexpressing 69Q huntingtin (Supplement Fig. 2 and 3).

**Fig. 3:**
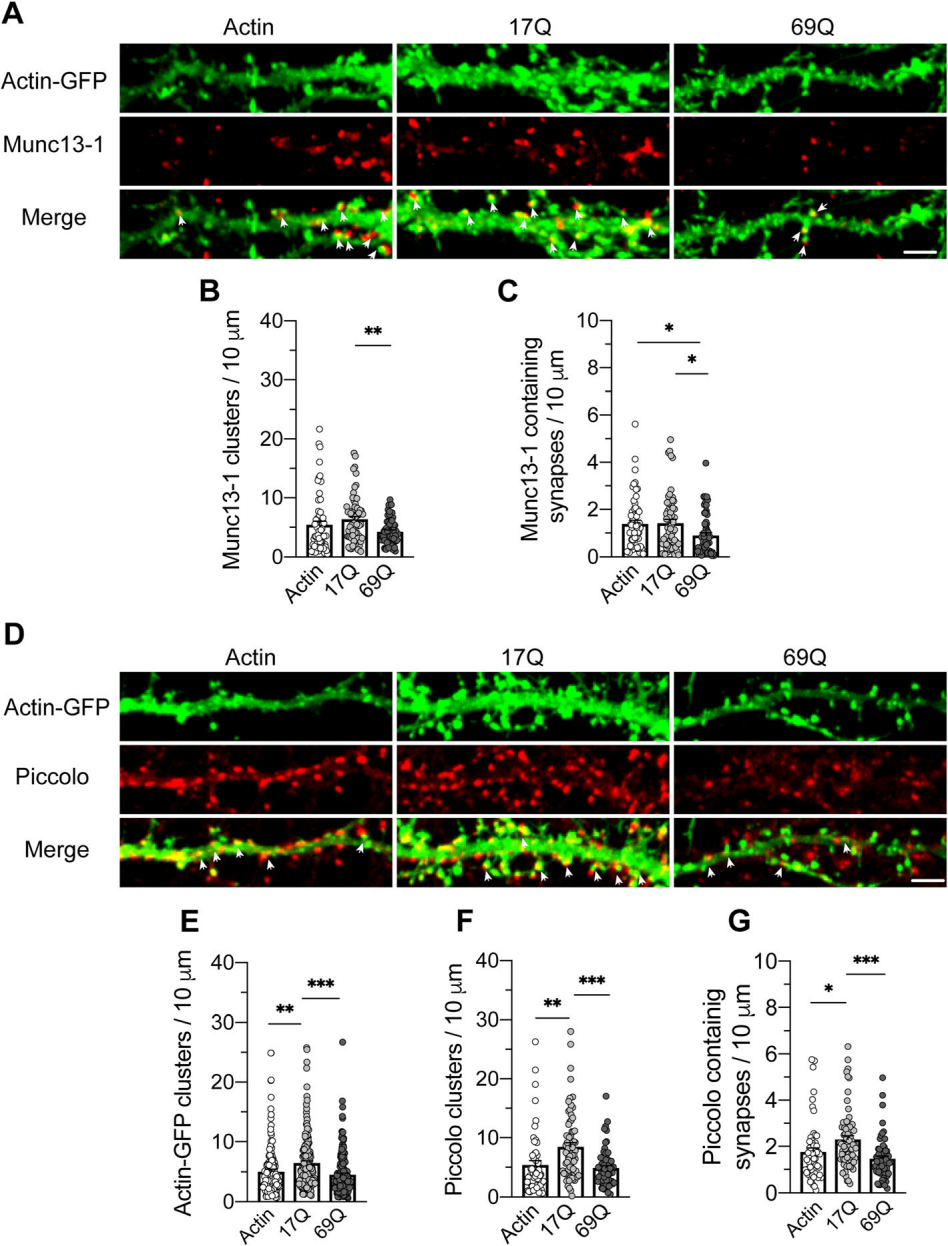
Effects of other active zone proteins (Piccolo and Munc13–1) in huntingtin overexpressing hippocampal neurons. **a** Immunofluorescence showed a notable loss of Munc13–1 clusters and Munc13–1 containing mature synapses (**a**, arrowheads) induced by mutant huntingtin (69Q) overexpression. The quantitative analysis of Munc13–1 (**b**) and its synapses (**c**), *n* = 61–64 segments/group, *N* = 21–22 cells/group. **d** 69Q huntingtin overexpressing resulted in a dramatic decrease of presynaptic bouton marked by Piccolo, dendritic spine labeled by Actin-GFP and mature synapses (**d**, arrowheads) compared to 17Q condition, while 17Q overexpressing promoted numbers of Actin, Piccolo clusters and mature synapses when compare with control condition (Actin-GFP group). The statistics of Actin clusters (**e**) Piccolo clusters (**f**) and Piccolo containing mature synapses (**g**). *n* = 56–70 segments/group, *N* = 19–23 cells/group. All data obtained by at least triplicate individual experiments, one way ANOVA test, followed by a Tukey’s multiple comparisons post-test, *p < 0.05, **p < 0.01, ***p < 0.001 (Scale bar = 5 μm)

When measuring the density of synapses, we found decreased numbers of presynaptic boutons marked by the Bassoon antibody, in neurons expressing 69Q huntingtin compared to cells expressing 17Q huntingtin (mean spine number/10 μm; 69Q: 4.25 ± 0.42; 17Q: 6.94 ± 0.77; actin-GFP: 5.53 ± 0.65; one-way ANOVA, *p* = 0.0088; Fig. 2b, c). Further, we counted the numbers of the mature synapses (pre-synapses marked by Bassoon adjacent to post-synapses marked by actin-GFP). Neurons overexpressing 69Q huntingtin exhibited a significant reduction of mature synapses compared to neurons expressing 17Q huntingtin or actin-GFP (mean mature synapse number/10um; 69Q: 1.35 ± 0.14; 17Q: 2.42 ± 0.23; actin-GFP: 2.01 ± 0.19; one-way ANOVA, *p* = 0.0002; Fig. 2b, d). A comparable reduction in spine density induced by 69Q huntingtin was seen for Munc13–1 (mean spine number/10 μm; 69Q: 4.27 ± 0.27; 17Q: 6.36 ± 0.50; actin-GFP: 5.42 ± 0.63; one-way ANOVA, *p* = 0.01; Fig. 3a, b). Similarly, a notable loss in the number of Munc13–1 containing mature synapses was observed in cells overexpressing 69Q huntingtin compared to 17Q huntingtin (mean mature synapse number/10 μm; 69Q: 0.907 ± 0.102; 17Q: 1.43 ± 0.15; actin-GFP: 1.40 ± 0.14; one-way ANOVA, *p* = 0.0073; Fig. 3a, c). Interestingly, Piccolo and actin positive profiles were equally reduced by overexpression of 69Q huntingtin compared to 17Q condition, but showing a promotion by 17Q overexpression when compared with actin-GFP condition (mean spine number/10 μm; 69Q: 4.89 ± 0.40; 17Q: 8.50 ± 0.68; actin-GFP: 5.410 ± 0.70; one-way ANOVA, *p* < 0.0001; Fig. 2a, 3a, d-g). In addition, the mature synapse numbers (pre-synapses marked by Piccolo) consistently showed the same tendency as Piccolo puncta (Mean mature synapse number/10 μm; 69Q: 1.47 ± 0.11; 17Q: 2.30 ± 0.16; actin-GFP: 1.76 ± 0.18; one-way ANOVA, *p* = 0.0003; Fig. 3d, g). These data indicate that 69Q huntingtin causes significant reductions of active zone proteins (especially Bassoon and Munc13–1) and dendritic spines [28, 29], which may eventually lead to loss of mature synapses.

### Loss of bassoon protein in human brains tissues

**Fig. 4:**
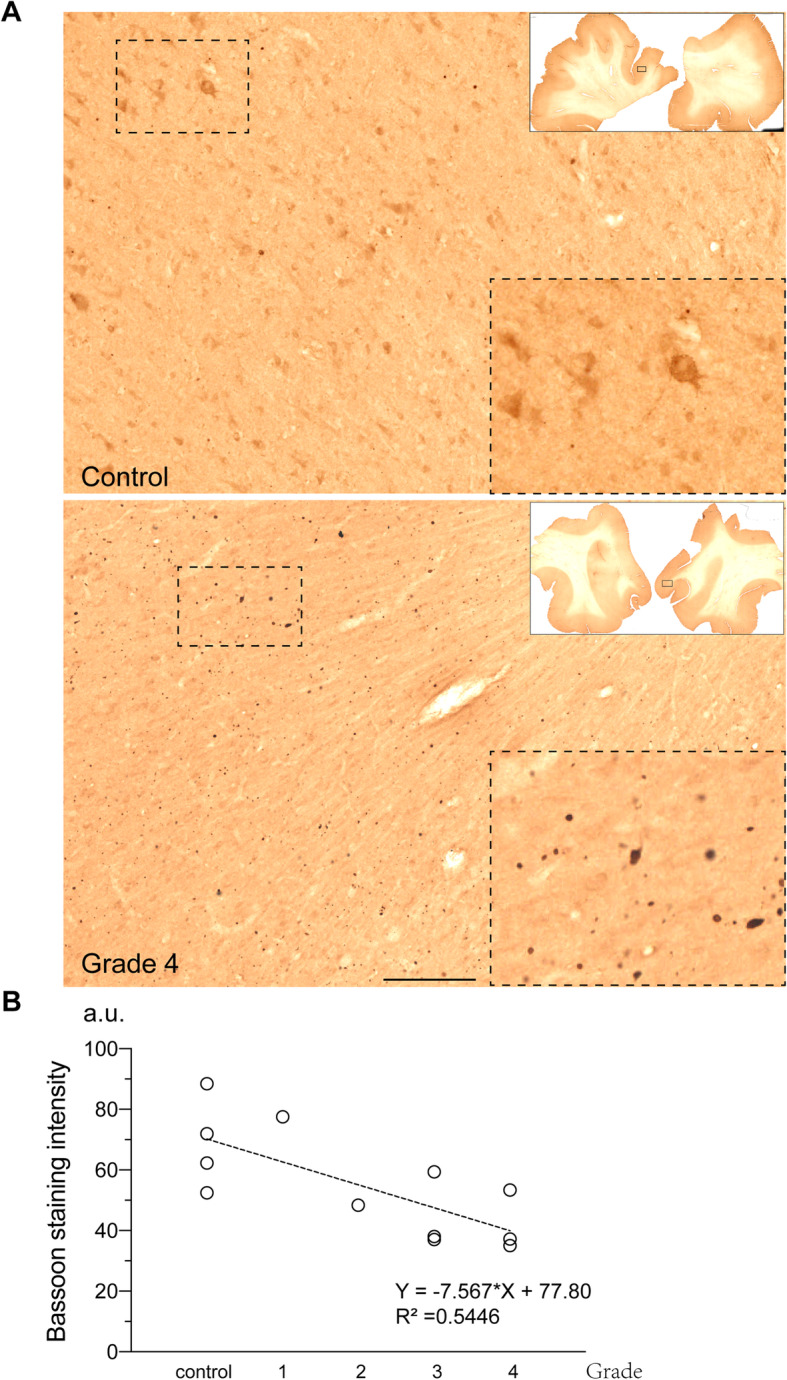
Immunohistochemistry of Bassoon in HD patient superior frontal gyrus. **a** DAB-stained sections from a control (upper) and a grade 4 patient (lower) brain (Scale bar = 200 μm). **b** Measurement of DAB-staining intensity. The signal in the gray matter of the cortex was measured (arbitrary units) and plotted against disease grades. Spearman correlation, tied *p* = 0.02

To study whether or not the reduction in Bassoon levels was restricted to the huntingtin overexpressing cells and HD mouse models, we studied the expression of Bassoon in the frontal cortex of HD patients and controls (Table 1). Using immunohistochemistry, we observed that control brains showed a diffuse cytoplasmic staining pattern for Bassoon (Fig. 4a, upper panel) while it was mainly localized in large aggregate-like structures in the deep cortical layers of HD brains (Fig. 4a, lower panel). To estimate levels of Bassoon in the HD patient brain, we measured the intensity of Bassoon staining in the cortex of HD patients and control subjects (Fig. 4b). There was a significant decrease in Bassoon levels in the cortex with increasing disease grade (Jonckheere-Terpstra Test, 2-tailed, *p* = 0.02).

### Bassoon is recruited to huntingtin aggregates in an age-dependent manner

**Fig. 5:**
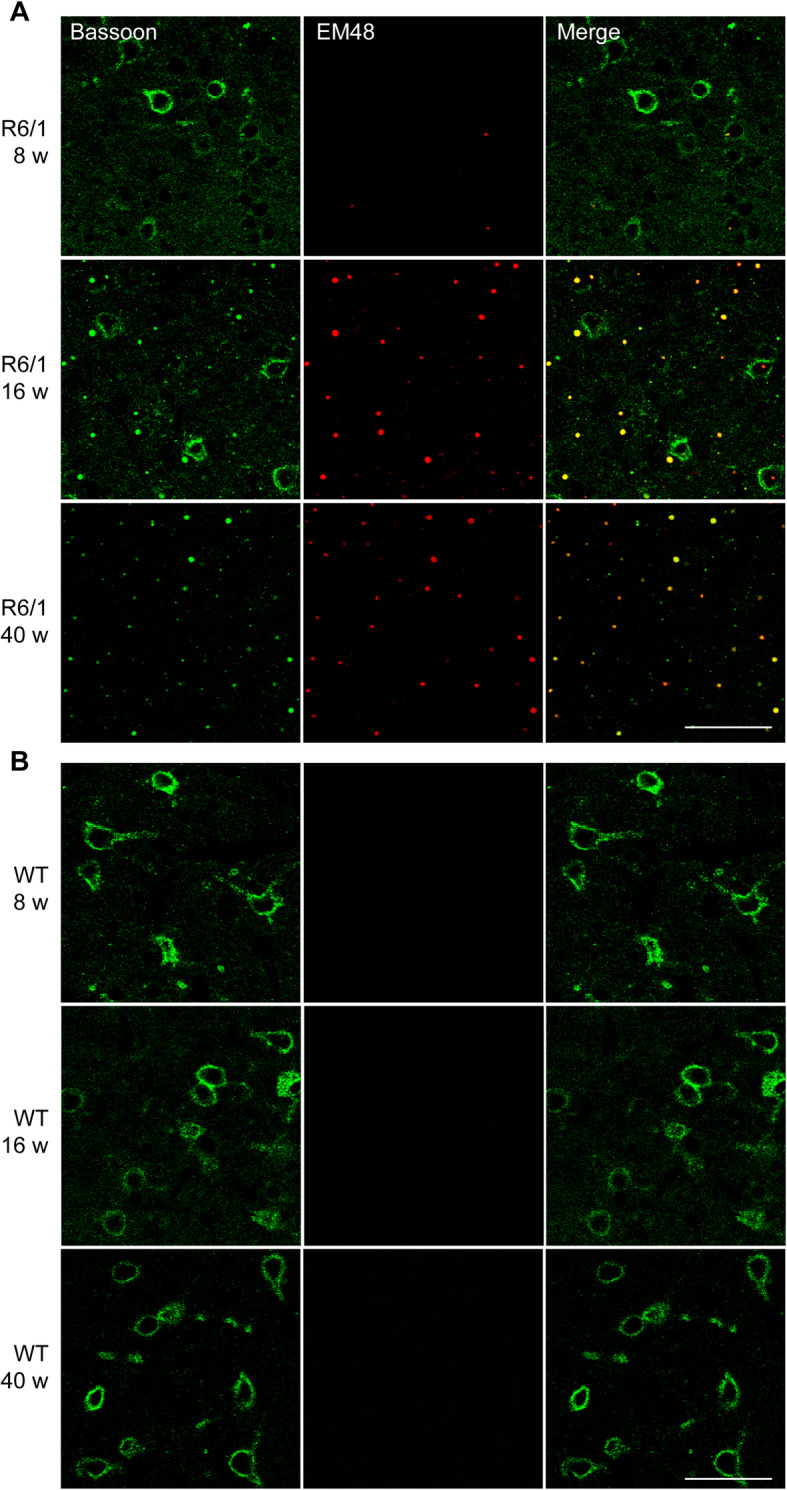
Immunohistochemistry of Bassoon in cortex of R6/1 and WT animals. **a** Double labeling of 8, 16, 40 weeks R6/1 cortex. EM48 positive aggregates are beginning to form at 8 weeks. In 40-week-old R6/1, inclusions are clear and there is a strong colocalization of Bassoon with the aggregates (scale bar = 20 μm). **b** Double labeling of 8, 16, 40 weeks WT cortex. No positive signal of EM48 exhibits and Bassoon staining maintains unchanged at different ages of mice (Scale bar = 20 μm)

**Fig. 6:**
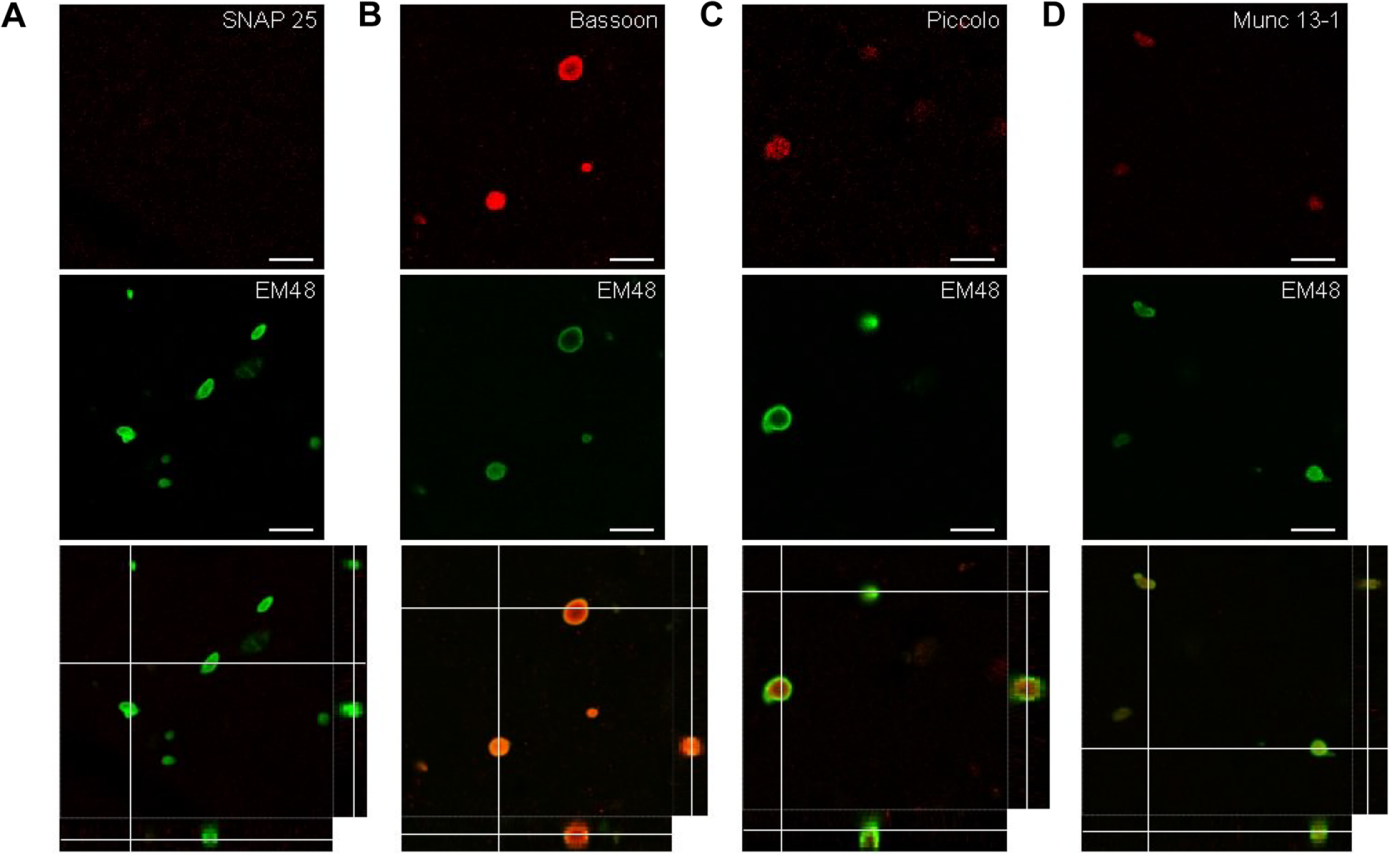
Double labeling of huntingtin inclusions and active zone proteins in HD patient cortex. Double immunofluorescence of huntingtin aggregates and SNAP-25 (**a**), Bassoon (**b**), Piccolo (**c**) and Munc 13–1 (**d**). The upper panels represent one confocal plane and the merged pictures (bottom panel) represent the merged pictures with the z-stacks shown on the sides (scale bars = 10 μm)

Our results have illustrated that active zone protein Bassoon exhibited a dramatic reduction in HD animal models, in primary neurons overexpressing mutant huntingtin and in brain tissue from HD patients. However, the mechanism underlying this reduction remains elusive. We next assessed the pattern of Bassoon expression in R6/1 and WT animals using immunohistochemistry (Supplement Fig. 4). The normal bassoon expression pattern is a punctuate synaptic staining combined with a diffuse cytoplasmic localization. Interestingly, we observed that the expression pattern was largely altered in the cortex of older R6/1 mice (Fig. 5, Supplement Fig. 4). In 8-week-old mice mutant huntingtin aggregates have begun to form, but the Bassoon expression pattern was largely unchanged (Fig. 5a, b top panels). Of note, the association between Bassoon and huntingtin inclusions was apparent already at the age of 16 weeks (Fig. 5a, b second panels) when the first motor symptoms appear in the R6/1 mouse model [10, 17, 22, 25, 46], implying the effect of colocalization induced the presynaptic dysfunction and led to locomotor impairment. At 40 weeks of age, the normal Bassoon signal was drastically reduced and the Bassoon protein seemed to be predominantly present in the mutant huntingtin aggregates (Fig. 5a, b bottom panels, Supplement Fig. 5A). We observed the majority of huntingtin aggregates overlapped with Bassoon positive punctae. More interestingly, the expression pattern of Bassoon in the striatum was similarly altered as in the cortex, even more sever (Supplement Fig. 5B). The association of Bassoon and huntingtin also existed in an age-dependent manner (Supplement Fig. 6A). Overall, these findings suggested that pathological processes which related to Bassoon and huntingtin interactions may be consistent in the cortex and striatum, these pathological changes eventually resulting in motor symptoms. Yet primary neurons expressing mutated huntingtin (69Q) did not have the same effect. The proteins in active zone did not form the aggregates even after neurons were mature. In addition, immunohistochemistry against Piccolo, Munc 13–1 and huntingtin aggregates similarly showed a gradual increase in Piccolo or Munc 13–1 and mutant huntingtin colocalization with increasing age (Fig. 6, Supplement Fig. 7).

### Bassoon and Huntingtin aggregate association in human tissues

To elucidate whether Bassoon was associated with huntingtin-containing inclusions also in the cortical brains tissues from patient with HD, we double-stained sections with antibodies against huntingtin aggregates (EM48) and SNAP25, Bassoon, Piccolo and Munc 13–1 (Fig. 6a-d). We observed a high degree of colocalization between the three active zone proteins Bassoon, Piccolo and Munc 13–1 and huntingtin aggregates (Fig. 6b-d). However, we never detected any colocalization between SNAP25, another presynaptic protein involved in vesicle exocytosis, and mutant huntingtin (Fig. 6a). Taken together, our data strongly support the claim that active proteins, especially Bassoon, associate with huntingtin aggregates, in both HD animal models and patients.

### Bassoon interacts with mutant huntingtin inclusions by an indirect interaction

**Fig. 7:**
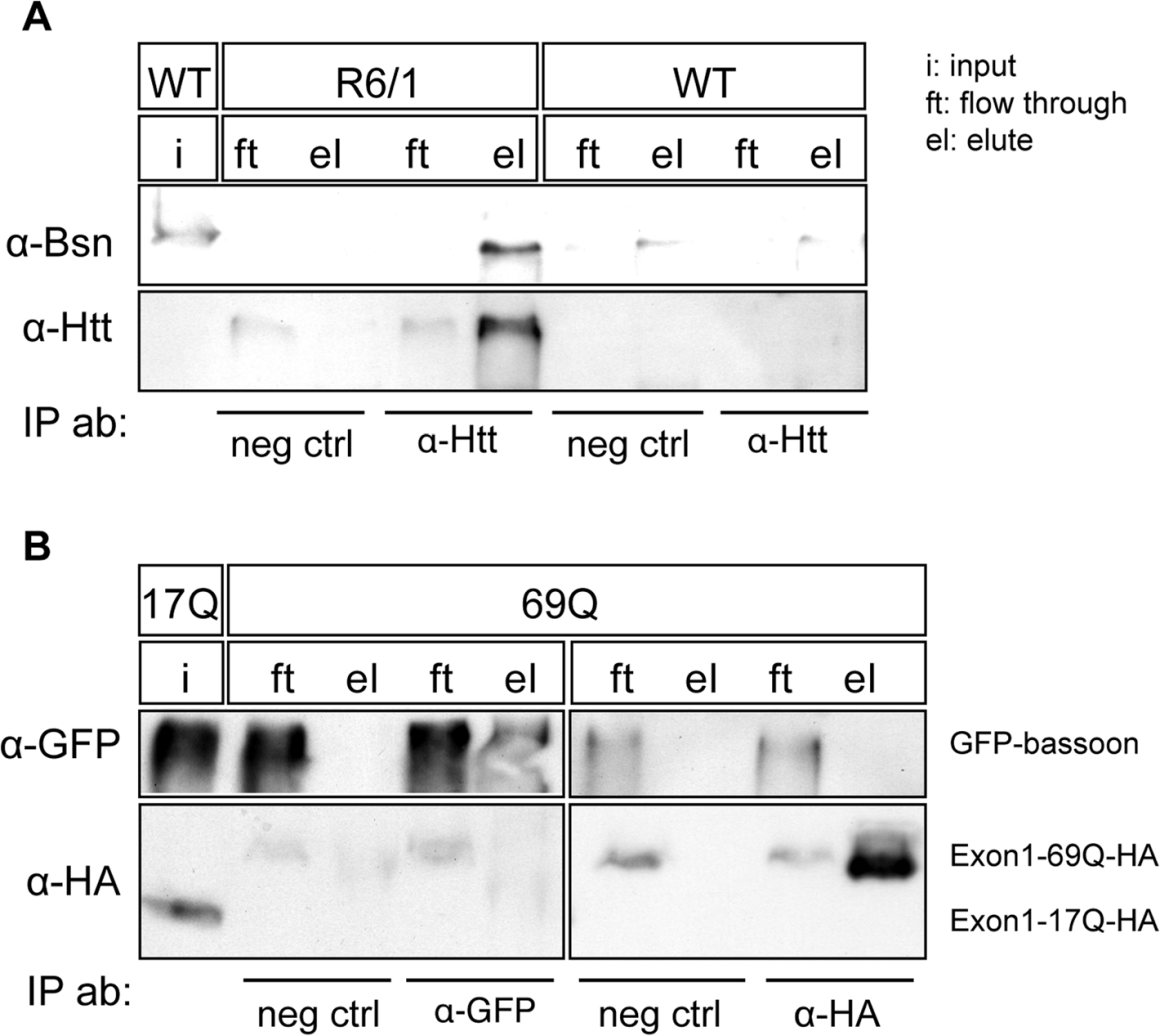
Immunoprecipitation of bassoon by mutated huntingtin. **a** Immunoprecipitation in R6/1 mice using a huntingtin antibody. The huntingtin antibody pulls down aggregated mutated huntingtin (lower panel) and bassoon (upper panel). **b** Immunoprecipitation in HEK 293 cells. (**b**, left panels) Precipitation using a GFP antibody pulls down GFP-Bassoon (upper panel), but fails to pull down HA-huntingtin (lower panel). (**b**, right panels) Immunoprecipitation using an HA antibody pulls down 69Q-HA-huntingtin (lower panel), but fails to pull down GFP-bassoon (upper panel)

The high degree of colocalization of Bassoon and mutant huntingtin in inclusions prompted us to evaluate whether Bassoon was passively sequestered into aggregates, or whether it undergoes an active recruitment by protein-protein interaction. To this end, we immunoprecipitated huntingtin using an antibody against N-terminal huntingtin. The antibody preferentially pulled down mutated huntingtin and huntingtin aggregates (Fig. 7a). When incubating the eluate with an antibody against Bassoon, we detected a prominent Bassoon band in the eluate, indicating that the pull down of huntingtin indeed also included Bassoon (Fig. 7a, upper panel). We next tested whether the interaction between Bassoon and huntingtin was a direct interaction or whether it occurs via a third linking protein. We therefore transiently transfected HEK 293 cells with GFP-Bassoon and either HA-17Q or HA-69Q huntingtin. We then pulled down Bassoon and huntingtin via their GFP and HA tags. Since the HEK 293 cells do not express high levels of active zone proteins, Bassoon and huntingtin will only be pulled down by the other partner if the interaction is direct. We could readily pull down both HA-huntingtin and GFP-Bassoon, using HA and GFP antibodies respectively, which confirmed there was no splicing for tag proteins. But neither huntingtin was pulled down with the GFP antibody nor Bassoon with the HA antibody (Fig. 7b). These results indicate that the interaction between Bassoon and huntingtin is mediated by a third linking protein.

## Discussion

The presynaptic active zone provides sites for vesicle docking and release at synapses and is essential for speed and accuracy of synaptic transmission. In the present study, we have investigated expression levels and patterns of proteins that constitute the cytomatrix of the active zone (CAZ) in mouse and cell culture models of HD and in postmortem brain tissues of HD patients. We observed a striking reduction and redistribution of three CAZ proteins (Bassoon, Piccolo and Munc 13–1) in cultured cells transfected with mutant (69Q) huntingtin, brains of R6/1 mice and patients with HD. In addition, the proteins were recruited into intranuclear and neurotic aggregates, away from their normal site of action at the presynaptic active zones. Moreover, we found an interaction between mutant huntingtin and Bassoon. This interaction does not seem to be direct, since it was not observed when huntingtin and Bassoon were overexpressed alone, in the absence of other interacting proteins.

### Reduced availability of active zone proteins

We found that the level of Bassoon was decreased in the cultured neurons transfected with mutant (69Q) huntingtin, in the R6/1 HD model and in the cortex of HD patients. We observed a concurrent decrease in mRNA levels of Bassoon in the R6/1 mice. Interestingly, we did not observe changes in the mRNA levels of the other active zone proteins. Decreased levels of striatal Bassoon mRNA have previously been reported in a large microarray study on HD patient samples [11]. Taken together, this suggests that the huntingtin mutation alters transcription of Bassoon and/or the half-life of its mRNA. Decreased availability of Bassoon in the presynaptic components could possibly affect synaptic functions. Importantly, we also found that three active zone proteins Bassoon, Piccolo and Munc 13–1 all translocated into the nucleus or distributed to intrasomal or neurotic huntingtin inclusions. Why and how these large proteins are transported into the nucleus remain obscure. The transport of the active zone proteins from Golgi apparatus to the presynaptic compartments has been suggested to occur in large Piccolo transport vesicles (PTVs) [7, 37]. The proteins forming the CAZ interact in large complexes [34]. Thus, if one of the proteins is trapped by aggregated huntingtin, the others may follow. Since active zone proteins are crucial for presynaptic vesicular release, loss of these functional complexes would be detrimental for synaptic function.

### Mechanisms of the interaction between bassoon and huntingtin

To address why the active zone proteins colocalize with the huntingtin aggregates, we searched for an interaction between huntingtin and Bassoon in immunoprecipitation experiments with huntingtin antibodies. We found that mutant, but not wild type, huntingtin pulled down Bassoon in mouse forebrain lysates. Interestingly, in rodents Bassoon has a polyglutamine stretch (11–24 glutamine residues) in the C-terminus which therefore may interact with other polyglutamine-containing proteins [44]. However, human Bassoon only contains 6 glutamine residues [50]. Using synaptosomal preparations from the cerebral cortex, striatum and hippocampus in combination with strategies of huntingtin antibody co-immunoprecipitation and nano LC-MS/MS, Yao et al. (2014) revealed that huntingtin is consistently associated with the presynaptic cytomatrix proteins Bassoon and Piccolo at physiological conditions [51]. To assess whether the interaction between huntingtin and Bassoon was direct or indirect, we overexpressed both proteins in HEK-cells. In this model Bassoon and WT/mutant huntingtin did not interact with each other. These data argue against a direct interaction between huntingtin and Bassoon. Therefore, it is conceivable that Bassoon may bind to huntingtin via an unidentified linker, e.g. Piccolo or Munc 13–1.

### Mechanisms underlying the deficiency of bassoon protein

In the present study, the high degree of colocalization between huntingtin intranuclear aggregates and Bassoon and Piccolo indicates that intranuclear re-distribution of these active zone proteins may be an active sequestration process, which will lead to relative depletion of the proteins in the pre-synaptic terminals and impair synaptic function. Moreover, several previous studies have illustrated that huntingtin plays a crucial role in postsynaptic function, such as regulating the trafficking or surface expressions of AMPA receptors and NMDA receptors in HD models [14, 38, 52]. We have previously reported a deficiency in spines resulting from mutant huntingtin expression [28, 29]. In addition to interactions within pre-synapse, we wondered whether the mutant huntingtin protein interrupted the active zone proteins from postsynaptic component. In in vitro experiments, overexpression of huntingtin (17Q or 69Q) only existed on transfected neurons and the surroundings were common and naive neurons. Thus, the aberrant alterations of those active zone proteins may be resulted from the abnormal postsynaptic huntingtin expression. Those results suggest that post-synaptic mutant huntingtin may disturb the active zone proteins through pre-and post-synaptic interactions. It gave rise to the idea that mutant huntingtin might have two pathways to modulate the synaptic components, one via the recruitments of presynaptic boutons, the other one through the interactions between pre- and post-synapses. Both mechanisms may contribute to the mutant huntingtin mediated synaptic dysfunctions and eventually lead to the loss of mature synapses.

### Functional consequences of decreasing bassoon expression

Based on the information from Bassoon knockout (KO) animals [1], selective knock-down of Bassoon does not induce drastic changes in the synaptic function. There are slight changes, however, with an increased proportion of the synapses being inactive in spite of a normal number of vesicles in the readily releasable pool of vesicles [1]. Interestingly, 50% of homozygous Bassoon KO animals die from epileptic seizures within the first 6 months. Epileptic seizures are also common features in the R6 mouse model of HD and in patients with juvenile HD [20]. In addition, mice where the Bassoon gene has been depleted exhibited impaired presynaptic function, sensory deficits, developed seizures, and displayed selectively enhanced contextual fear memory and increased novelty preference [2]. In Bassoon KO animals, Piccolo is up-regulated to 1.4 times normal levels, potentially compensating for some of the lost Bassoon function [1]. Interestingly, elimination of Piccolo by using RNAi caused enhanced synaptic vesicle exocytosis rate and synaptic vesicle recycling, but had no effects on formation of glutamatergic synapses [21]. Similar phenomenon was not seen after Bassoon knock out. This implies, that although a protein complex of Bassoon and Piccolo is normally formed, functionally the proteins have related, distinct roles in synaptic function. In HD mice and patients, however, both Bassoon and Piccolo are recruited to the huntingtin inclusions. Therefore, the full functional protein-complex is reduced at the active zone, possibly leading to a more severe functional deficit than that caused by an isolated loss of a single protein.

## Conclusion

In summary, we observed a progressive decrease in the levels of the active zone protein Bassoon in mouse and cell culture models of HD and in brain tissues of patients with HD. Moreover, we report that the active zone proteins Bassoon, Piccolo and Munc 13–1 are redistributed from the presynapse to huntingtin aggregates. Taken together, these changes may underlie synaptic functional defects and cause the synaptic loss observed in late stage HD. These data suggest that loss of active zone proteins may contribute to the pathogenesis of HD and serve as biomarkers of synaptopathology.

## Supplementary information

**Additional file 1: Figure S1.** Quantification of protein levels of active zone and related proteins in the cortex and the striatum of 40 weeks old R6/1 and WT mice. In the cortex and striatum, no clear changes of active zone proteins CASK (A), Mint1 (B), Rim1 (C) and ERC (D), except Bassoon (F) and Munc13–1 (E).

**Additional file 2: Figure S2.** Immunostaining of Bassoon and mutant Huntingtin protein in transfected primary culture. Neurons were co-transfected with actin-GFP (upper panel) and 17Q huntingtin (Middle panel) or 69Q huntingtin (lower panel). Huntingtin aggregates were clearly labeled by EM48 antibody in the cell body of neurons expressing mutant huntingtin (69Q) (Lower panel). In addition, the level of Bassoon was decreased in neurons expressing mutant huntingtin (69Q), compared to the neurons with control (GFP alone) plasmid or 17Q huntingtin expression (scale bar = 50 μm).

**Additional file 3: Figure S3.** Triple labeling of Munc13–1, huntingtin inclusion, and actin-GFP in primary culture. Neurons were co-transfected with actin-GFP (upper panel) and 17Q huntingtin (Middle panel) or 69Q huntingtin (lower panel). There is a clear inclusion of huntingtin appearing in the neurons with mutant huntingtin (69Q) expression (Lower panel), whereas the level of Munc13–1 was decreased. Scale bar = 50 μm.

**Additional file 4: Figure S4.** Immunohistochemistry of Bassoon in the cortex of 8, 16 and 40 weeks old R6/1 and WT mice. The appearance of aggregates of Bassoon correlates with the age of disease onset. Arrowheads point to Bassoon positive cell bodies, and aggregates (40w of R6/1 mouse). Scale bar = 50 μm.

**Additional file 5: Figure S5.** Immunohistochemistry of Bassoon and huntingtin in the cortex and striatum of 16 weeks old R6/1 and WT animals**.** (A) High magnification z-stacks through a huntingtin positive inclusion in the cortex of R6/1 mice (scale bar = 10 μm). (B) Double labeling of 16 weeks R6/1 (1st panel) and WT (2nd panel) cortex (scale bar = 75 μm). Huntingtin inclusions are clear and colocalize with Bassoon aggregates in both the cortex and striatum at 16 weeks of R6/1 mice.

**Additional file 6: Figure S6.** Immunohistochemistry of Bassoon in the striatum of 8 and 40 weeks old R6/1 and WT mice. (A) Double labeling of 8 weeks R6/1 (1st panel) and WT (2nd panel) striata. EM48 positive aggregates are beginning to form. 40-week-old R6/1 (3rd panel) and WT (4th panel) striata. Inclusions are evident and there is a high colocalization of Bassoon aggregates with the huntingtin inclusions (scale bar = 50 μm). (B) High magnification z-stacks through a huntingtin positive inclusion (left) from a R6/1 mouse and a Bassoon positive WT neuron (right). Scale bar = 10 μm).

**Additional file 7: Figure S7.** Immunohistochemistry of Piccolo and Bassoon in the cortex and striatum of R6/1 and WT animals at age of 40 weeks. Piccolo shows some aggregate formation in the cortex and striatum of aged R6/1 mice (40 weeks). Similarly, Bassoon inclusions were observed abundantly in both regions of R6/1 mice. Scale bars = 100 μm in low magnified images, 20 μm in inlets.

## Data Availability

All data generated or analyzed during this study are included in this published article (and its supplementary information files).

## Abbreviations

17Q: Huntingtin with 17 glutamine residues
69Q: Huntingtin with 69 glutamine residues
BA9: Brodmann area 9
CAZ: Cytoskeletal matrix of the active zone
CMV: Cytomegalovirus
DAB: Diaminobenzidine
DIV: Days in vitro
KO: Knockout
N/A: Not available
PFA: Paraformaldehyde
PTVs: Piccolo transport vesicles
SFG: Superior frontal gyrus
SNAP25: Synaptosomal nerve-associated protein 25
SNARE: Soluble NSF Attachment Protein Receptor
