# Supplementary figures and images for: No symphony without bassoon and piccolo: changes in synaptic active zone proteins in Huntington’s disease
Source: Acta Neuropathol Commun. 2020 Jun 3;8:77. doi: 10.1186/s40478-020-00949-y (PMC7268643; doi:10.1186/s40478-020-00949-y)

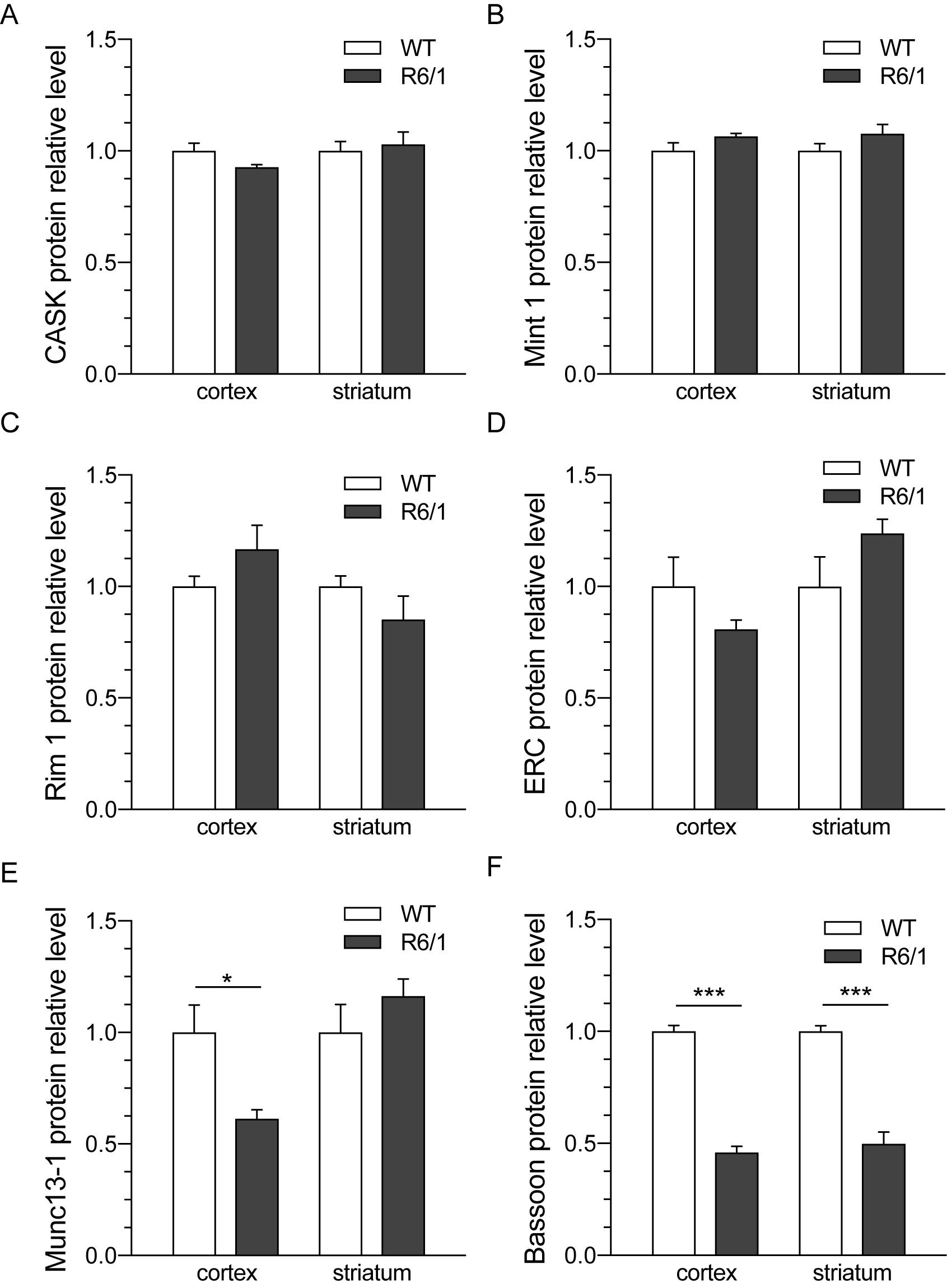

Supplement: Supplementary file 1 — Additional file 1: Figure S1. Quantification of protein levels of active zone and related proteins in the cortex and the striatum of 40 weeks old R6/1 and WT mice. In the cortex and striatum, no clear changes of active zone proteins CASK (A), Mint1 (B), Rim1 (C) and ERC (D), except Bassoon (F) and Munc13–1 (E). [file 40478_2020_949_MOESM1_ESM.tif]

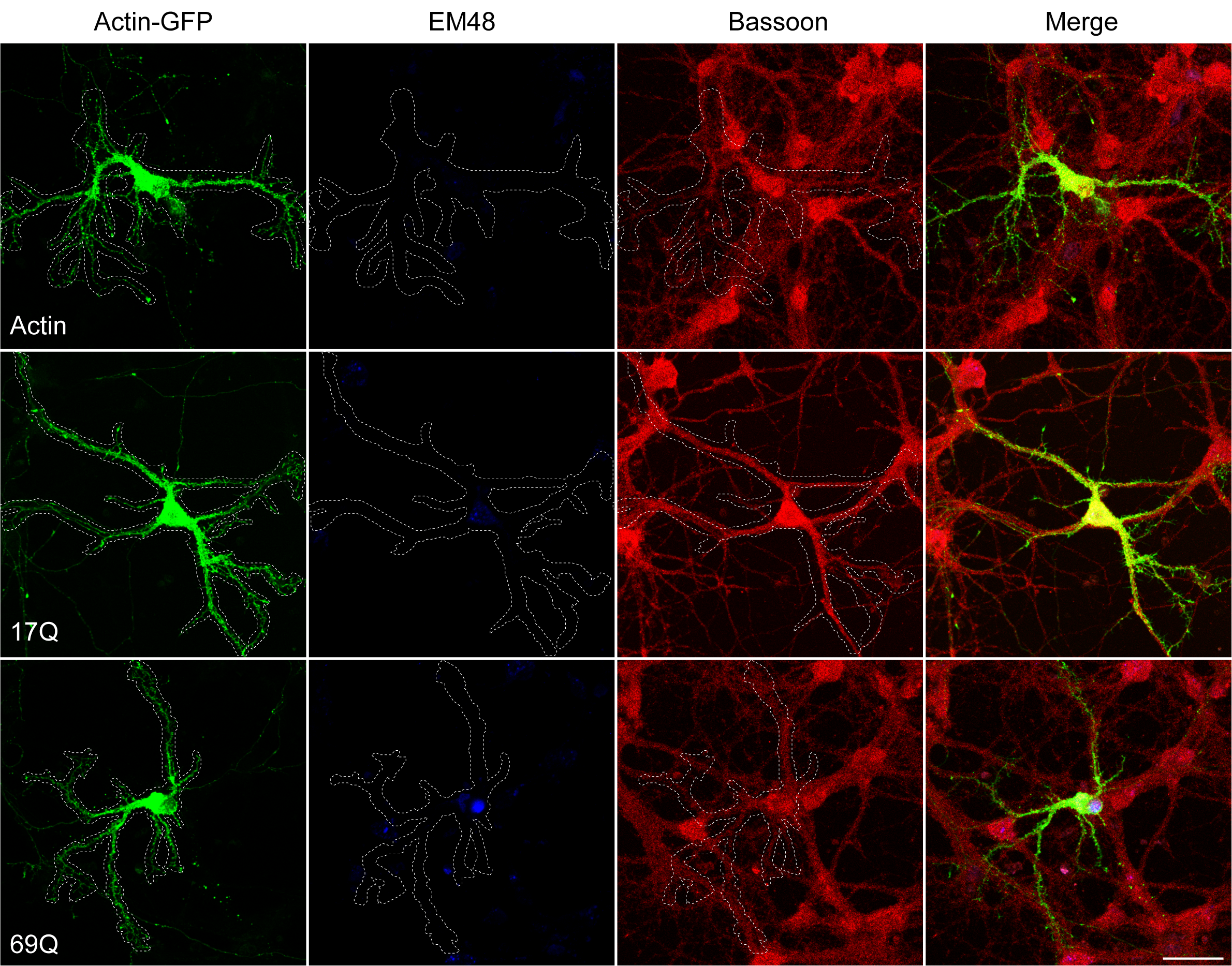

Supplement: Supplementary file 2 — Additional file 2: Figure S2. Immunostaining of Bassoon and mutant Huntingtin protein in transfected primary culture. Neurons were co-transfected with actin-GFP (upper panel) and 17Q huntingtin (Middle panel) or 69Q huntingtin (lower panel). Huntingtin aggregates were clearly labeled by EM48 antibody in the cell body of neurons expressing mutant huntingtin (69Q) (Lower panel). In addition, the level of Bassoon was decreased in neurons expressing mutant huntingtin (69Q), compared to the neurons with control (GFP alone) plasmid or 17Q huntingtin expression (scale bar = 50 μm). [file 40478_2020_949_MOESM2_ESM.tif]

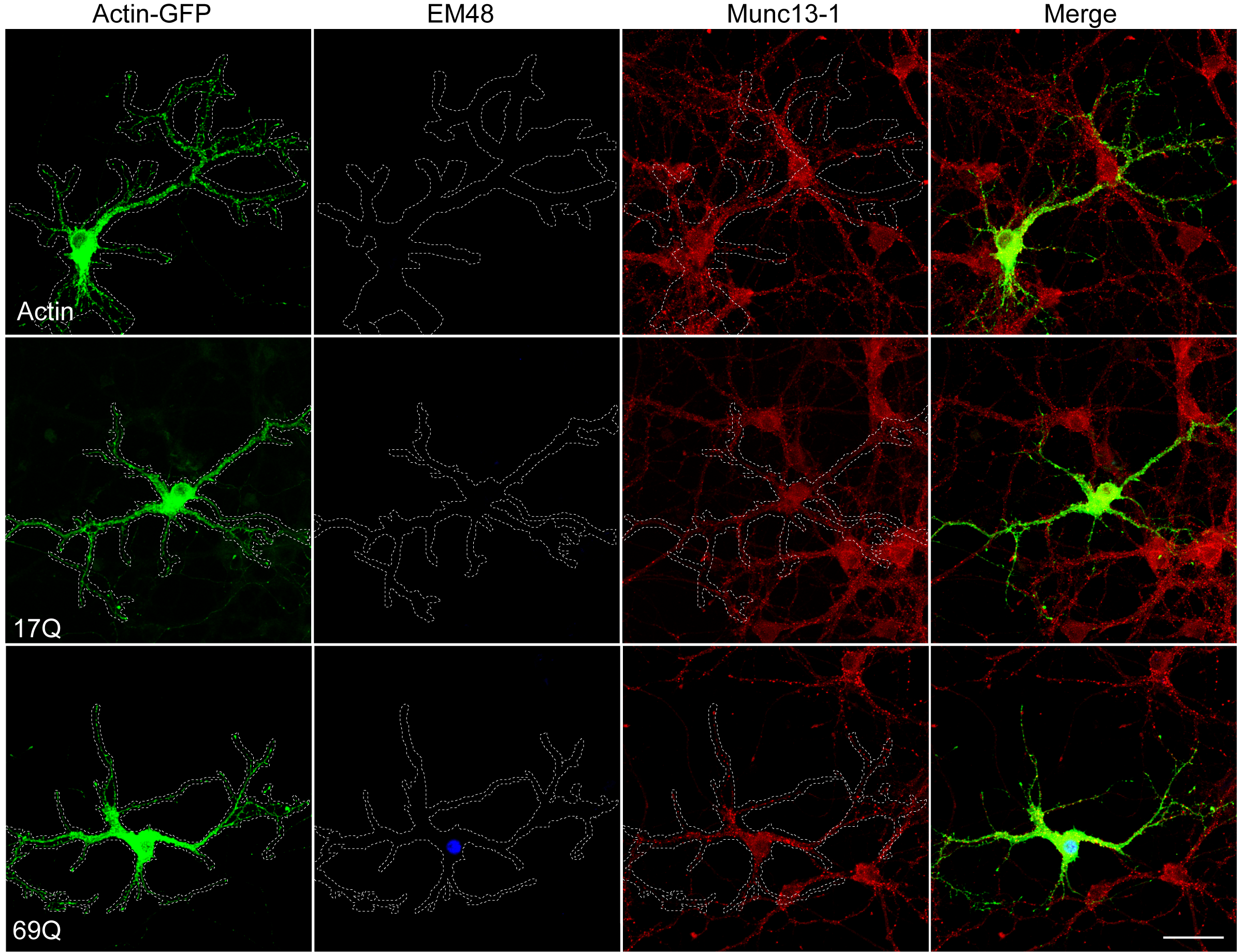

Supplement: Supplementary file 3 — Additional file 3: Figure S3. Triple labeling of Munc13–1, huntingtin inclusion, and actin-GFP in primary culture. Neurons were co-transfected with actin-GFP (upper panel) and 17Q huntingtin (Middle panel) or 69Q huntingtin (lower panel). There is a clear inclusion of huntingtin appearing in the neurons with mutant huntingtin (69Q) expression (Lower panel), whereas the level of Munc13–1 was decreased. Scale bar = 50 μm. [file 40478_2020_949_MOESM3_ESM.tif]

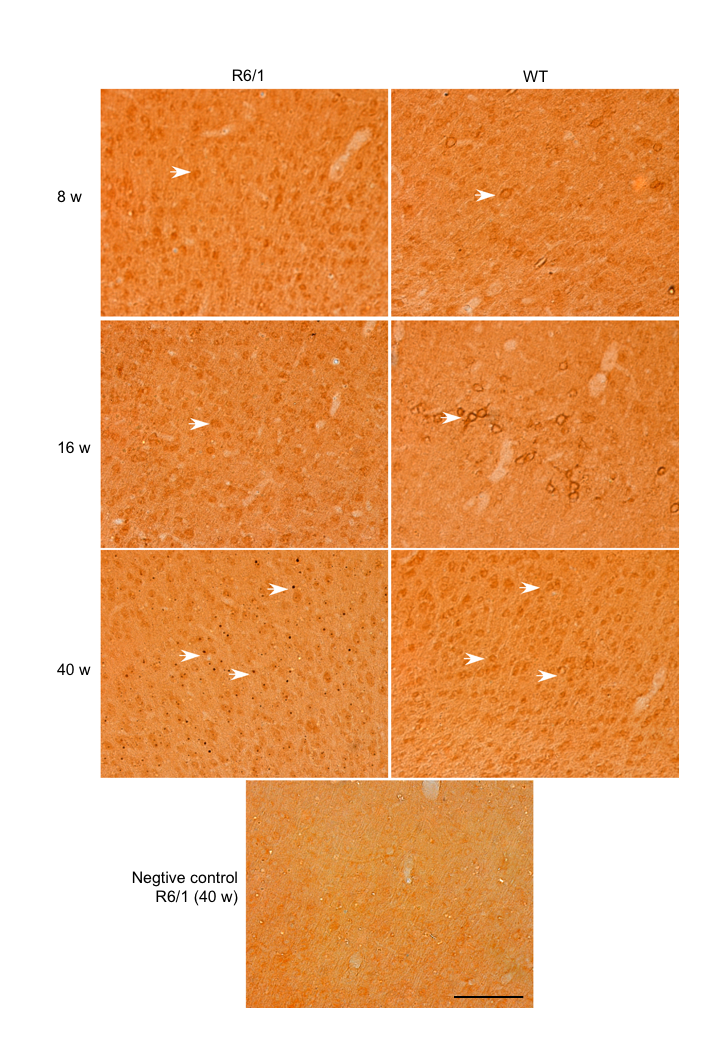

Supplement: Supplementary file 4 — Additional file 4: Figure S4. Immunohistochemistry of Bassoon in the cortex of 8, 16 and 40 weeks old R6/1 and WT mice. The appearance of aggregates of Bassoon correlates with the age of disease onset. Arrowheads point to Bassoon positive cell bodies, and aggregates (40w of R6/1 mouse). Scale bar = 50 μm. [file 40478_2020_949_MOESM4_ESM.tiff]

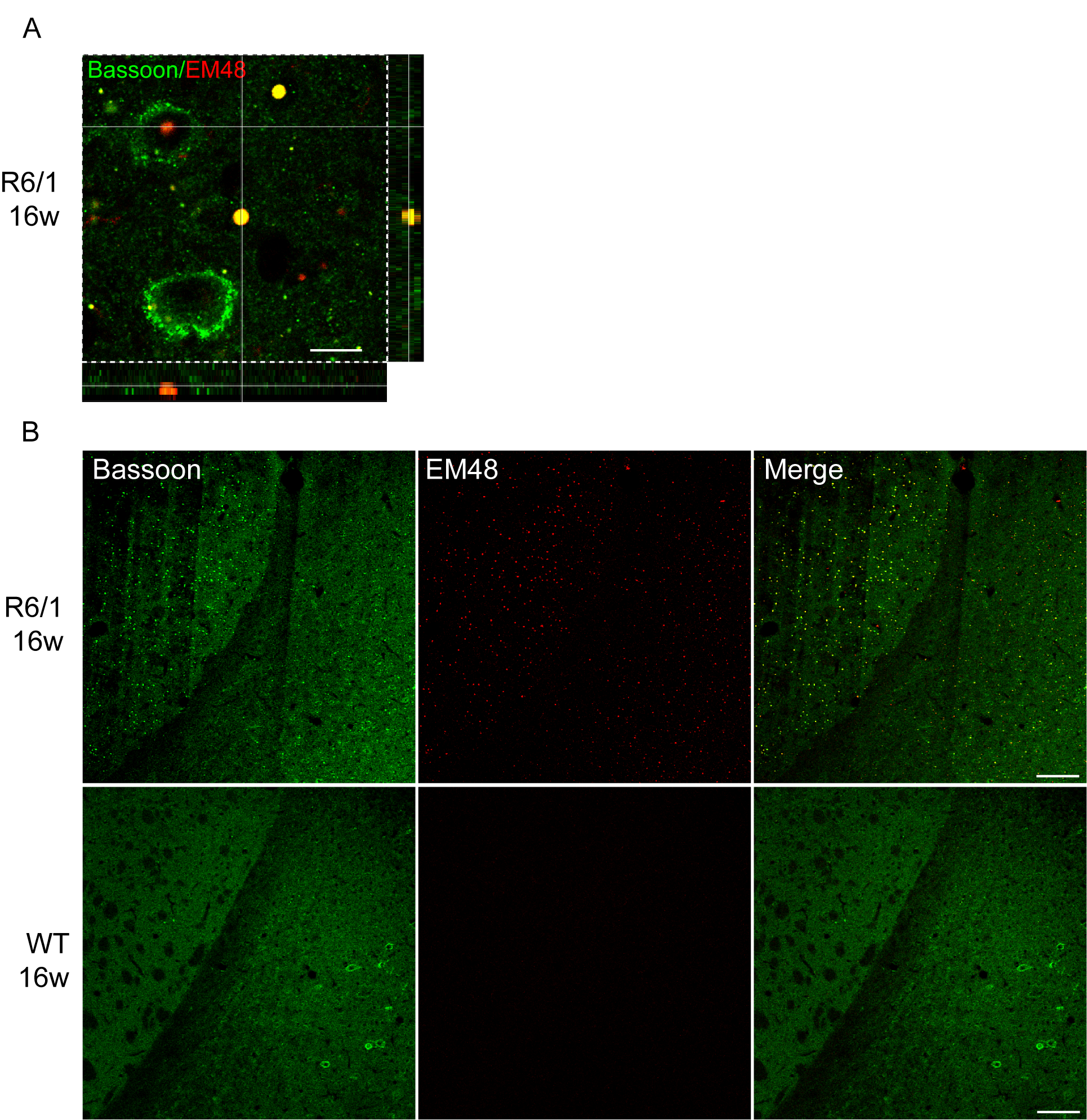

Supplement: Supplementary file 5 — Additional file 5: Figure S5. Immunohistochemistry of Bassoon and huntingtin in the cortex and striatum of 16 weeks old R6/1 and WT animals. (A) High magnification z-stacks through a huntingtin positive inclusion in the cortex of R6/1 mice (scale bar = 10 μm). (B) Double labeling of 16 weeks R6/1 (1st panel) and WT (2nd panel) cortex (scale bar = 75 μm). Huntingtin inclusions are clear and colocalize with Bassoon aggregates in both the cortex and striatum at 16 weeks of R6/1 mice. [file 40478_2020_949_MOESM5_ESM.tif]

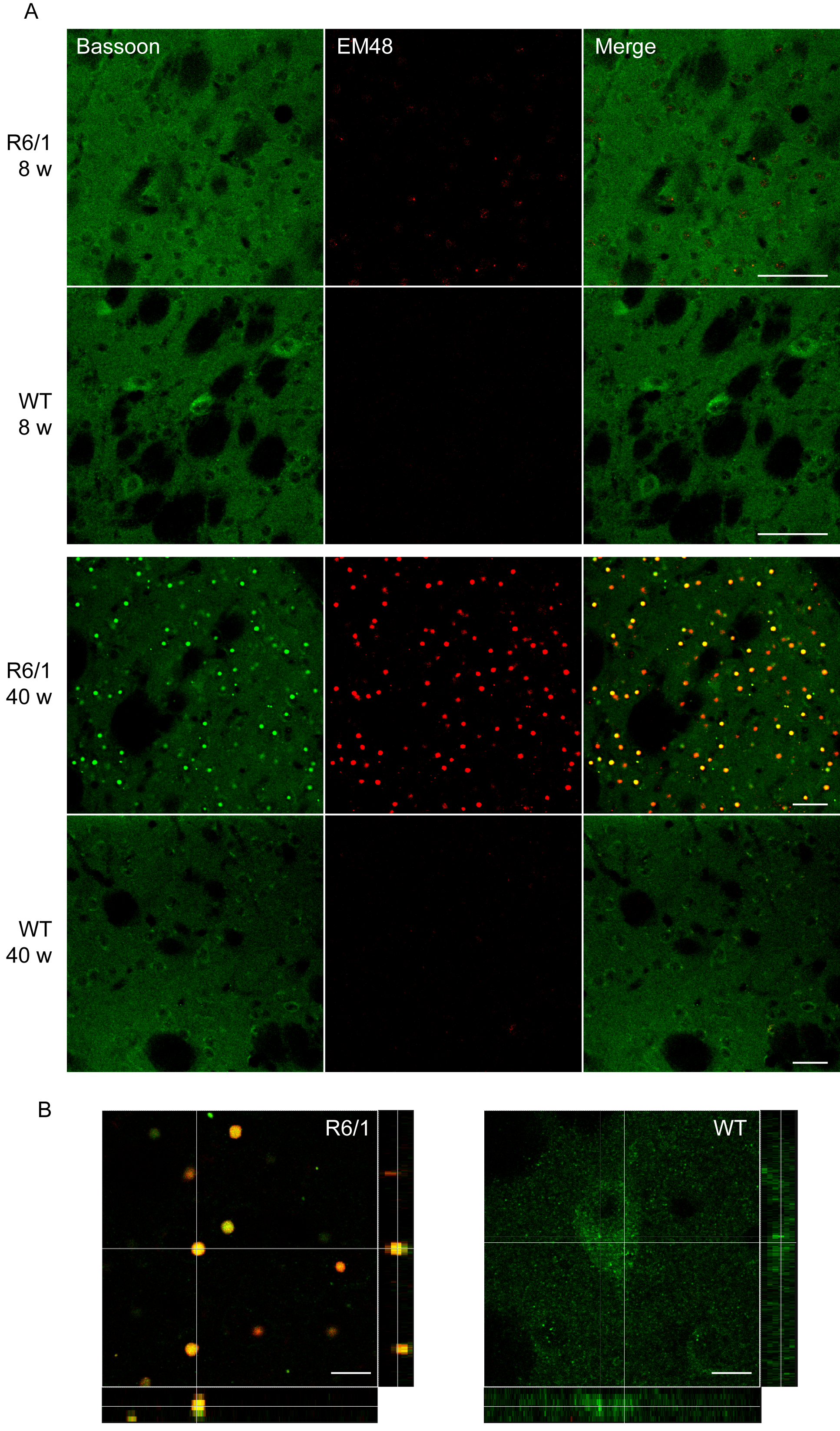

Supplement: Supplementary file 6 — Additional file 6: Figure S6. Immunohistochemistry of Bassoon in the striatum of 8 and 40 weeks old R6/1 and WT mice. (A) Double labeling of 8 weeks R6/1 (1st panel) and WT (2nd panel) striata. EM48 positive aggregates are beginning to form. 40-week-old R6/1 (3rd panel) and WT (4th panel) striata. Inclusions are evident and there is a high colocalization of Bassoon aggregates with the huntingtin inclusions (scale bar = 50 μm). (B) High magnification z-stacks through a huntingtin positive inclusion (left) from a R6/1 mouse and a Bassoon positive WT neuron (right). Scale bar = 10 μm). [file 40478_2020_949_MOESM6_ESM.tif]

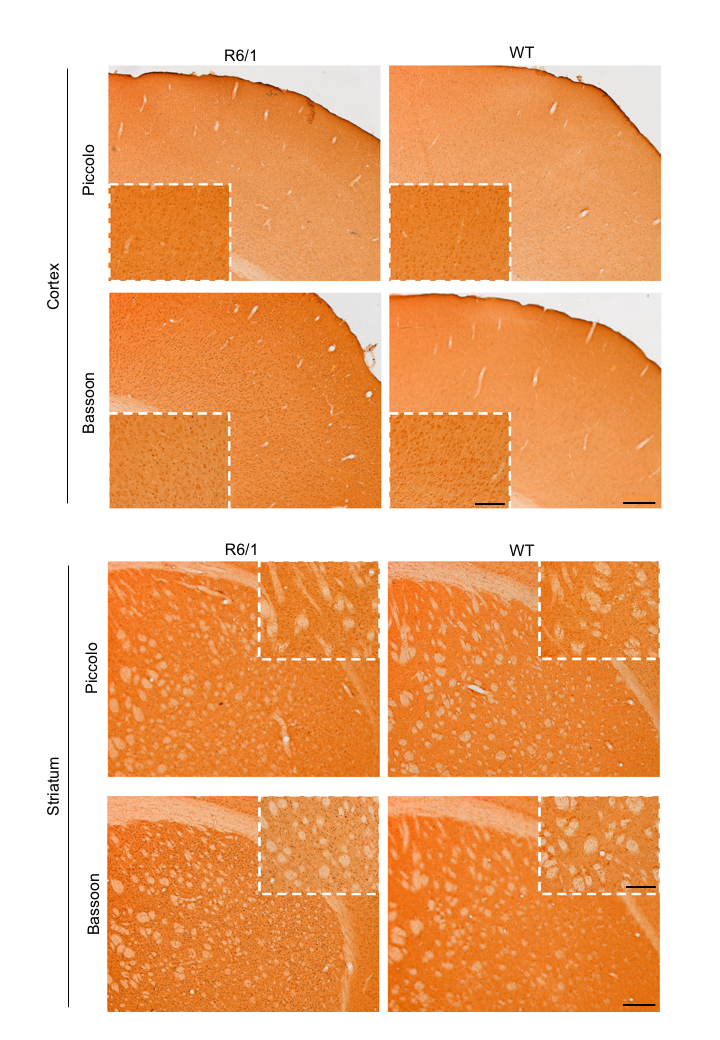

Supplement: Supplementary file 7 — Additional file 7: Figure S7. Immunohistochemistry of Piccolo and Bassoon in the cortex and striatum of R6/1 and WT animals at age of 40 weeks. Piccolo shows some aggregate formation in the cortex and striatum of aged R6/1 mice (40 weeks). Similarly, Bassoon inclusions were observed abundantly in both regions of R6/1 mice. Scale bars = 100 μm in low magnified images, 20 μm in inlets. [file 40478_2020_949_MOESM7_ESM.tiff]
